# Dengue Virus 2 NS2B Targets MAVS and IKKε to Evade the Antiviral Innate Immune Response

**DOI:** 10.4014/jmb.2210.10006

**Published:** 2023-02-15

**Authors:** Ying Nie, Dongqing Deng, Lumin Mou, Qizhou Long, Jinzhi Chen, Jiahong Wu

**Affiliations:** 1 The First Affiliated Hospital, Guizhou University of Traditional Chinese Medicine, Guiyang 550002, Guizhou Province, P.R. China; 2 Department of Parasitology; Provincial Key Laboratory of Modern Pathogen Biology, College of Basic Medical Sciences, Guizhou Medical University, Guiyang, 550025, P.R. China

**Keywords:** DENV2, NS2B, MAVS, IKKε, Innate immune response

## Abstract

Dengue virus (DENV) is a widespread arbovirus. To efficiently establish infection, DENV evolves multiple strategies to hijack the host innate immune response. Herein, we examined the inhibitory effects of DENV serotype 2 (DENV2) nonstructural proteins on RIG-I-directed antiviral immune response. We found that DENV2 NS2A, NS2B, NS4A, and NS4B significantly inhibited RIG-I-mediated IFN-β promoter activation. The roles of NS2B in RIG-I-directed antiviral immune response are unknown. Our study further showed that NS2B could dose-dependently suppress RIG-I/MAVS-induced activation of IFN-β promoter. Consistently, NS2B significantly decreased RIG-I- and MAVS-induced transcription of *IFNB1*, *ISG15*, and *ISG56*. Mechanistically, NS2B was found to interact with MAVS and IKKε to impair RIG-I-directed antiviral response. Our findings demonstrated a previously uncharacterized function of NS2B in RIG-I-mediated antiviral response, making it a promising drug target for anti-DENV treatments.

## Introduction

Dengue viruses, members of the family *Flaviviridae*, can be spread to humans from mosquitos. DENVs consist of four popularly known serotypes (DENV1-4) and cause about 390 million people to be infected each year globally, of which 96 million cases are clinically manifested [1]. DENV infection results in a variety of diseases, ranging from mild fever to severe syndromes, such as dengue hemorrhagic fever and dengue shock syndrome [2]. To date, no antiviral drugs have been approved and no reliable, effective vaccine has been used for the therapy and prophylaxis of DENV [3-5].

**Fig. 1:**
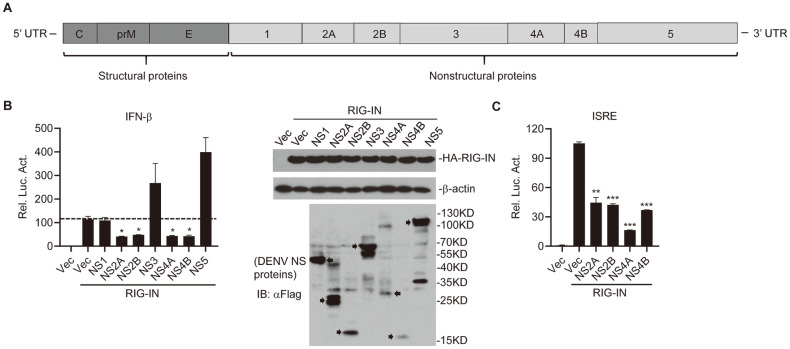
Inhibition of RIG-I-mediated activation of IFN-β and ISRE by DENV2 nonstructural proteins. (**A**) Schematic of DENV2 encoded proteins. The polyprotein is proteolytically cleaved into 3 structural proteins (C, prM, and E) and 7 nonstructural (NS) proteins (NS1, NS2A, NS2B, NS3, NS4A, NS4B, and NS5) by viral and cellular proteases. (**B**) Effects of DENV2 nonstructural proteins on RIG-I-mediated activation of IFN-β promoter. HEK293T cells were cotransfected with IFN-β or ISRE reporter (0.05 μg), pRL-TK (0.05 μg), empty vectors, or viral protein expression plasmids (0.05 μg) together with indicated RIG-IN expression plasmids (0.1 μg). Twenty-four hours after transfection, cells were lysed for luciferase assays. In the right panel, expression of DENV NS proteins, RIG-IN, and β-actin were assessed by western blot assay. (**C**) NS2A, NS2B, NS4A, and NS4B inhibit RIG-I-mediated ISRE activation. The experiments were similarly performed as in B. Graphs show mean ± SD. *n* = 3. *p < 0.05, **p < 0.01, ***p < 0.001 (Student’s *t*-test).

Dengue virus possesses a ~10.7 kb, single-stranded positive-sense RNA genome. Translation of the genome produces a polyprotein (Fig. 1A), which is further processed by proteases into three structural proteins (C, E, and prM), which are the component of the virion particle, and seven nonstructural (NS) proteins (NS1, NS2A, NS2B, NS3, NS4A, NS4B, and NS5) which are involved in multiple processes such as polyprotein cleavage, viral replication, and regulation of host immune response [6].

Viral infections are first recognized and fought by the innate immune system. During DENV infection, both RIG-I-like receptors (RLRs) and Toll-like receptors (TLRs) are involved in DENV recognition [7, 8]. RIG-I and MDA5, members of RLRs, sense and bind to DENV viral RNA in the cytosol and then translocate to mitochondria, where they bind and activate their common adaptor MAVS (alternatively referred to VISA, IPS-1, and Cardif) [9-12]. MAVS recruits and activates kinases, including TBK1 and IKKε. Subsequently, the transcription factors IRF3 and NF-κB are phosphorylated and activated, causing the expression of type I interferons (IFNs) [13]. The binding of IFNs with type I interferon receptors (IFNARs) activates the JAK-STAT pathway, producing numerous ISGs with antiviral functions [14, 15].

Several DENV nonstructural proteins have been evidenced to thwart the host IFN system. NS2A and NS4B from DENV4 significantly decreased RIG-I/MAVS/TBK1-directed activation of IFN-β and ISRE reporters[16]. NS4A disrupted RIG-I-MAVS interaction by targeting MAVS[17]. DENV NS2B associates with NS3 to form NS2B/3 protease and is required for the NS3-carrying protease activity [18]. NS2B/3 was found to interact with IKKε to mask the protein kinase domain, thereby blocking RLR signaling [19]. In addition, NS2B/3 and its protease cofactor NS2B prevent cGAS-induced signaling transduction by binding to STING and cGAS, respectively [20-22]. Moreover, NS2A, NS4A, NS4B, and NS5 inhibit IFNa/b induced STAT1/2 activation that suppresses ISGs expression [23-26].

In this study, to further elucidate uncharacterized DENV interferon antagonists, we expressed all DENV2 nonstructural proteins with IFN-β luciferase reporters, along with plasmids expressing RIG-IN (constitutively active mutant of RIG-I, containing two CARD domains) [27]. This led us to identify NS2A, NS2B, NS4A and NS4B as antagonists of RIG-I-induced antiviral immunity. NS2A markedly suppressed RIG-IN-mediated IFN-β and ISRE activation, while NS2B, NS4A, and NS4B significantly inhibited RIG-IN and MAVS-mediated IFN-β and ISRE reporter activation. As far as we know, no reports have been published on how NS2B regulates RIG-I-directed antiviral response. Our further study showed that NS2B targets MAVS and IKKε, causing RIG-I-mediated signaling to be interrupted. These findings shed new light on our understanding of the immune evasion strategies utilized by DENV.

## Materials and Methods

### Reagents, Antibodies, Cells, and Virus

Lipo293 Transfection Reagent (Beyotime Biotechnology, C0521, China), dual-luciferase assay kits (Promega, E1910, USA). Flag-tag antibody (Sigma, F1804, USA), HA-tag antibody (Cell Signaling Technology, #2367, USA), and GAPDH antibody (Proteintech, 60004-1-Ig, China). HEK293T cells (CRL-3216) were purchased from ATCC (USA). DENV2 New Guinea C (NGC) strain was a gift from Prof. Tongyan Zhao (Beijing Institute of Microbiology and Epidemiology, China).

### Plasmid Construction

Plasmids for luciferase reporters (IFN-β and ISRE) and HA-tagged RIG-IN (residues 1 to 284), MAVS, TBK1, IRF3 were previously described [28]. pcDNA3.1 -IKKε-HA was purchased from Miaoling Plasmid (China). cDNA encoding all nonstructural proteins were amplified from DENV2 NGC strain cDNA and inserted into pCMV6-Entry Mammalian Expression Vector with C-terminal Flag tag (OriGene, USA) with standard molecular biology methods.

### Transfection and Dual Luciferase Assays

These experiments were performed as follows: HEK293T cells (1 × 10^5^) were seeded on 48-well plates. On the following day, cells were transfected with IFN-β or ISRE firefly reporter plasmid (50 ng/well), pRL-TK Renilla control reporter plasmid (50 ng/well), along with the indicated viral expression constructs using Lipo293 reagent for 24 h. Then, cells were harvested for dual luciferase assays.

### Quantitative Real-Time PCR

Cells were lysed in TRIzol reagent (ABP Biosciences, FP312, USA) for total RNA isolation. cDNA was synthesized with RT master mix reagent (MCE, HY-K0511A, USA) and changes of gene expression were observed by SYBR Green qPCR master mix (MCE, HY-K0501A, USA). The relative abundances of targeted genes were normalized to the values of GAPDH. Primers used in this research were described in the reference [28].

### Coimmunoprecipitation and Western Blot Analysis

These experiments were conducted as in the previous study [29]. Briefly, for Co-IP, the transient transfected HEK293T cells were lysed. The lysates were incubated with Flag antibody or control IgG, as well as protein G-Sepharose beads (GE Healthcare) for three hours. After that, the beads were washed. The precipitates and whole cell lysates were blotted with antibodies against Flag or HA.

### Statistics

Statistical analysis was conducted with GraphPad Prism 8.0.2 software. The unpaired student’s *t*-test was used to assess the significance (*p* < 0.05).

## Results

### DENV2 Nonstructural Proteins Antagonize RIG-I-Directed Pathway Responses

To examine DENV2 nonstructural proteins that may antagonize RIG-I-directed induction of IFN-β, we constructed all seven DENV2 nonstructural protein expression clones (Fig. 1A). Dual luciferase assays were performed to determine whether DENV nonstructural proteins affect RIG-I-directed transcription of IFN-β. Individual viral protein and IFN-β luciferase reporters were coexpressed in HEK293T cells with empty vectors or plasmids expressing RIG-IN. In the results, NS2A, NS2B, NS4A, and NS4B markedly suppressed the transcription of IFN-β mediated by RIG-IN (Fig. 1B). This was confirmed by the ISRE reporter assay showing that NS2A, NS2B, NS4A, and NS4B significantly inhibited ISRE luciferase activities directed by RIG-IN (Fig. 1C). Taken together, the above data suggested that these DENV2 encoded proteins act as antagonists of RIG-I-directed antiviral immune response.

### DENV2 NS2B, NS4A, and NS4B Significantly Inhibit RIG-I-Directed Signaling Activation at the MAVS Level

**Fig. 2:**
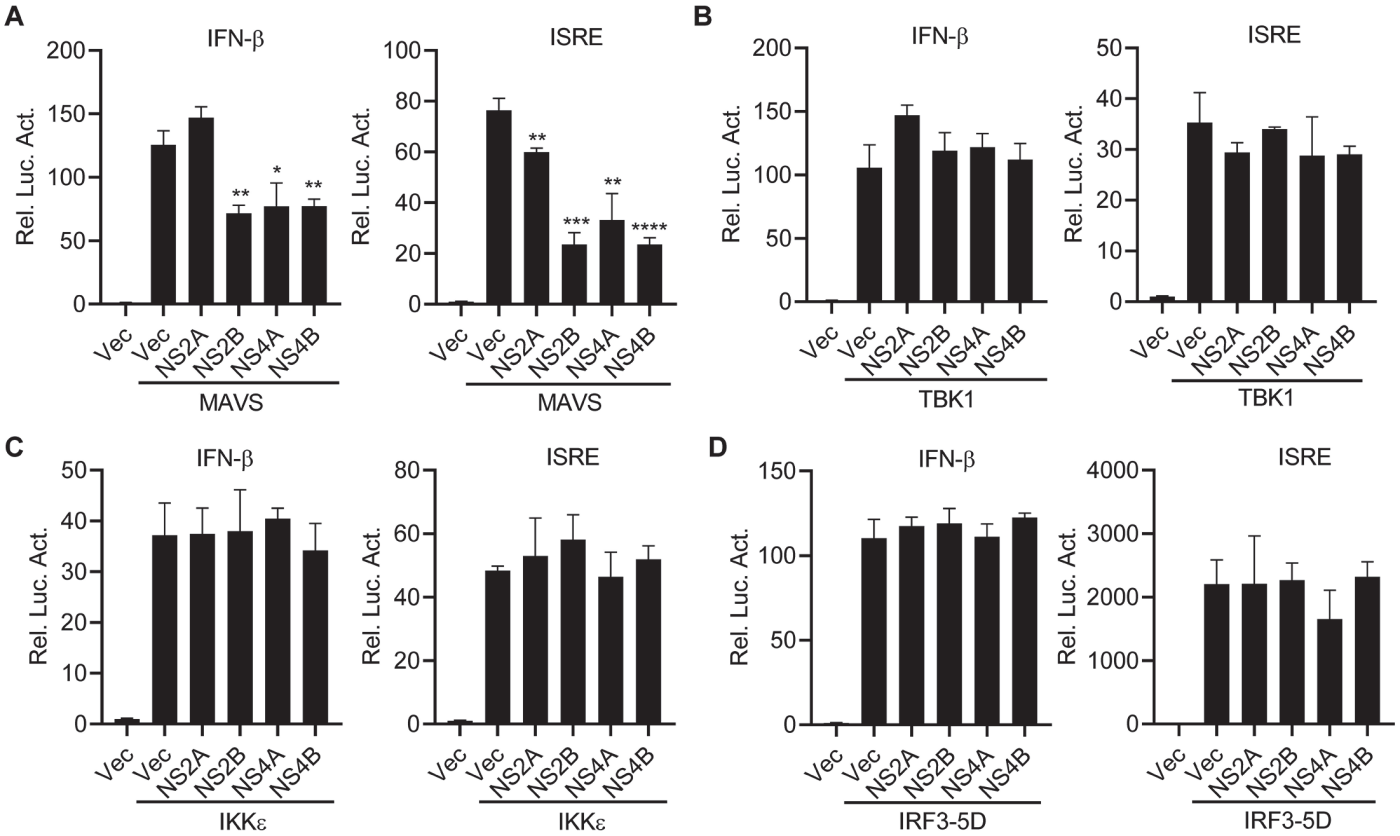
Effects of DENV2 proteins on MAVS, TBK1, IKKε, and IRF3-5D mediated activation of IFN-β transcription. HEK293T cells were cotransfected with IFN-β or ISRE reporter (0.05 μg), pRL-TK (0.05 μg), empty vectors or viral protein expression plasmids (0.05 μg) together with indicated MAVS (**A**), TBK1 (**B**), IKKε (**C**), and IRF3-5D (**D**) expression plasmids (0.05 μg). Twenty-four hours after transfection, cells were lysed for luciferase assays. Graphs show mean ± SD. *n* = 3. *p < 0.05, **p < 0.01, ***p < 0.001, ****p < 0.0001 (Student’s *t*-test).

MAVS, the common downstream adapter of RLRs, activates TBK1 and IKKε kinase, prompting the phosphorylation of IRF3 and transcription from IFN-β promoter [30]. To determine which DENV2 proteins can thwart IFN-β and ISRE activities mediated by MAVS, TBK1, IKKε and IRF3, MAVS, TBK1, IKKε or IRF3-5D (constitutively active form of IRF3) expression plasmids were cotransfected with individual viral gene, IFN-β or ISRE reporter plasmids. Curiously, NS2A inhibited MAVS-directed ISRE activation but did not interfere with MAVS-induced transcription of the IFN-β promoter (Fig. 2A). Intriguingly, we found that NS2B, NS4A, and NS4B significantly inhibit MAVS-, but not TBK1-, IKKε- or IRF3-5D-directed transcription of IFN-β and ISRE promoters, suggesting that NS2B, NS4A and NS4B inhibit RIG-I-induced antiviral response at the MAVS level (Figs. 2A-2D).

### DENV2 NS2B Inhibits RIG-I/MAVS-Induced Antiviral Response

NS2B acts as a cofactor of dengue NS2B/NS3 protease, which has been regarded as an essential target for anti-DENV drug discovery [18, 31]. A previous study has identified that NS2B targets and degrades DNA sensor cGAS to evade innate immune response [21]. However, its role in RIG-I-induced antiviral immune response has yet to be unveiled.

**Fig. 3:**
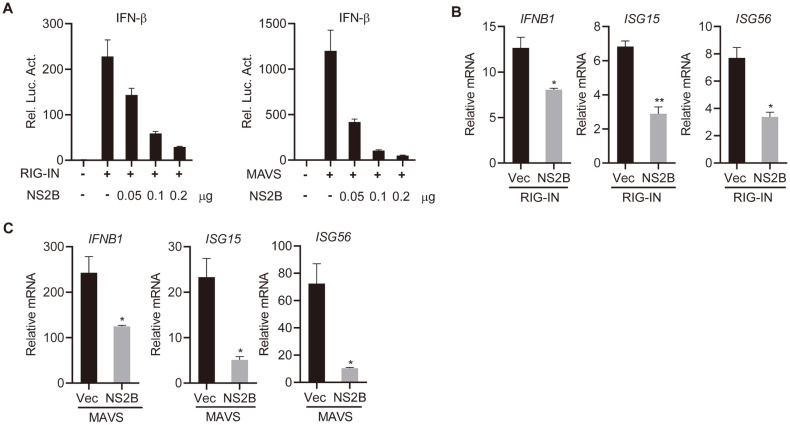
NS2B inhibits RIG-I- and MAVS-mediated downstream signaling activation. (**A**) NS2B dosedependently inhibits RIG-I- and MAVS-mediated activation of IFN-β promoter. HEK293T cells were cotransfected with empty vectors or increased amounts of NS2B expression plasmids, IFN-β (0.05 μg) and pRL-TK (0.05 μg) plasmids, along with RIG-IN or MAVS expression plasmids. Twenty-four hours after transfection, cells were lysed for luciferase assays. (**B** and **C**) NS2B inhibits RIG-I- and MAVS- directed transcription of *IFNB1*, *ISG15*, and *ISG56*. HEK293T cells were cotransfected with empty vectors or NS2B expression plasmids (1 μg), together with RIG-IN or MAVS expression plasmids. Twenty-four hours later, total RNA was isolated and the mRNA levels of indicated genes were analyzed by qPCR. Graphs show mean ± SD. *n* = 3. *p < 0.05, **p < 0.01, ***p < 0.001, ****p < 0.0001 (Student’s *t*-test).

To deeply define the roles of NS2B in RIG-I-directed antiviral response, cells were cotransfected with gradient doses of NS2B, IFN-β reporter plasmids, along with RIG-IN or MAVS expression plasmids. Results demonstrated that NS2B could dose-dependently reduce RIG-I- and MAVS-induced transcription of IFN-β promoter (Fig. 3A). Consistently, the qRT-PCR results revealed that overexpression of NS2B in cells dramatically downregulated RIG-I- and MAVS-directed induction of *IFNB1*, *ISG15*, and *ISG56* (Figs. 3B and 3C).

### DENV2 NS2B Interacts with MAVS and IKKε

**Fig. 4:**
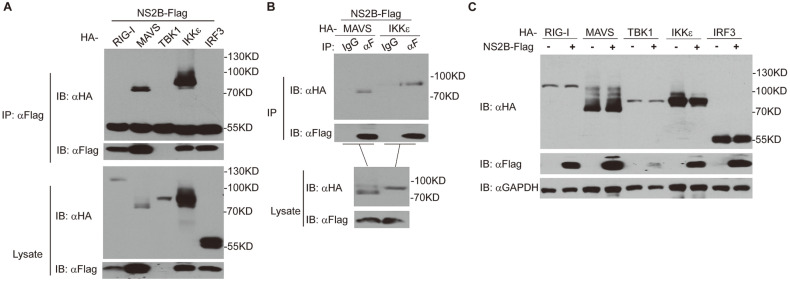
NS2B interacts with MAVS, IKKε. (**A** and **B**) NS2B targets MAVS and IKKε. HEK293T cells were transfected with indicated expression plasmids for 24 h, and co-immunoprecipitation and western blot were performed with the indicated antibodies. (**C**) Effects of NS2B on the protein expression levels of RLR signaling pathway components. HEK293T cells were cotransfected with HA-tagged components of the RLR signaling pathway, and vectors or NS2B-Flag for 24 h. The western blot analysis was performed with indicated antibodies.

Since NS2B inhibits RIG-I-induced antiviral signaling, we next determine which signaling component (s) is (are) targeted by NS2B. HEK293T cells transfected with NS2B-Flag and various HA-tagged components of RIG-I signaling were analyzed by coimmunoprecipitation (Co-IP). The results revealed that NS2B could strongly associate with MAVS and IKKε but not with other components of this pathway, including RIG-I, TBK1, and IRF3 (Fig. 4A). Additionally, Co-IP with IgG control further confirmed that NS2B targets MAVS and IKKε (Fig. 4B). Considering that NS2B degrades cGAS [21], we then investigate whether NS2B degrades MAVS and IKKε. Western blot analysis showed that NS2B does not affect the protein levels of MAVS, IKKε, and other key RLR signaling molecules (Fig. 4C). Collectively, these data demonstrated that NS2B targets MAVS and IKKε, resulting in the suppression of RIG-I-directed antiviral response.

## Discussion

During coevolution with hosts, DENVs evolved several ways to hijack the innate antiviral response [8]. In the current research, we aimed to show which DENV2 NS proteins modulate RIG-I-triggered transcription of IFN-β promoter. Results from dual luciferase assay demonstrated that NS2A, NS2B, NS4A, and NS4B function as negative modulators of the RLR signaling. NS2A downregulated RIG-IN-directed IFN-β and ISRE luciferase reporter activation, whereas NS2B, NS4A, and NS4B reduced RIG-IN- and MAVS-directed IFN-β and ISRE luciferase activities. Moreover, NS2B dose-dependently decreased MAVS-triggered transcription of IFN-β promoter and downregulated RIG-I and MAVS-directed the expression of downstream antiviral genes. Mechanistically, NS2B interacts with MAVS and IKKε, blocking RIG-I-mediated antiviral response.

Upon DENV infection, RLRs signal through downstream adaptor MAVS to initial antiviral response. MAVS forms polymers and serves as a platform to recruit TRAF proteins and TBK1/IKKε. In the platform, TBK1/ IKKε phosphorylates MAVS. Active MAVS then recruits IRF3 to its signalosome, where TBK1/IKKε phosphorylates and activates IRF3 [13, 32-34]. The crucial roles of MAVS in the RLRs signaling pathway have made it an attractive target for viruses [35, 36]. Many non-structural proteins of flaviviruses have been shown to target MAVS to avoid the host’s innate antiviral response. For example, Zika virus (ZIKV) NS3 and NS4A bind MAVS, thus blocking RLR-MAVS signaling [37-39]. Hepatitis C virus (HCV) directly cleaves mitochondrial and peroxisomal MAVS by its protease NS3/4A, thereby suppressing MAVS-induced immune response [40-43]. The new and emerging avian flavivirus Tembusu virus (TMUV) hinders host antiviral response by degrading MAVS via its NS2B [44]. NS4A of DENV is capable of binding the CARD domain and transmembrane domain of MAVS, which interrupts the association between RIG-I and MAVS [17]. Herein, we found that NS2B of DENV inhibits RLR signal transduction by targeting MAVS and IKKε. Moreover, NS2B does not downregulate both MAVS and IKKε protein expression (Fig. 4). However, NS2B inhibits MAVS- but not IKKε-mediated signaling (Fig. 2). This might be due to the interaction of NS2B with MAVS and IKKε interrupting the interaction of MAVS with IKKε, which impairs the activities of MAVS but not IKKε. In addition, as shown in Figs. 4A and 4C, when cotransfected with TBK1, expression of NS2B was markedly reduced. Therefore, we speculated that TBK1 might be a critical host factor against DENV infection by decreasing the expression of NS2B. Further studies are necessary to address these issues.

**Fig. 5:**
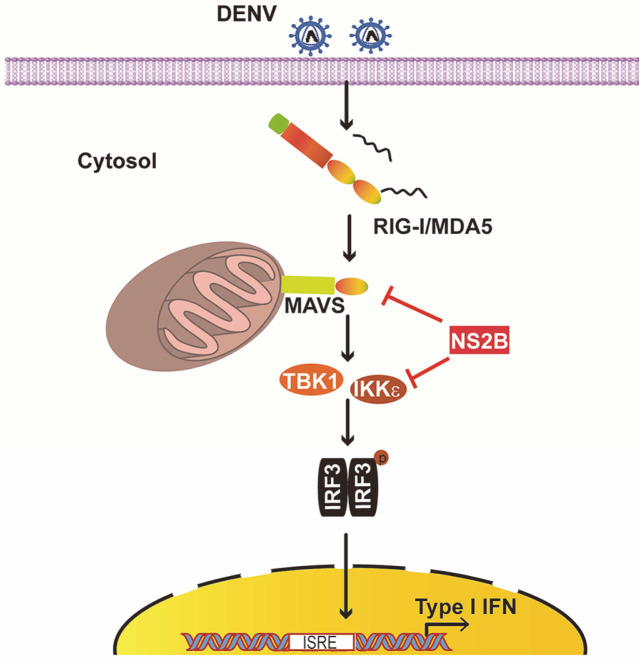
A working model for DENV2 NS2B antagonizes RLRs-mediated innate antiviral response. NS2B interacts with MAVS and IKKε, thus blocking the antiviral innate immune response.

In summary, our findings reveal that DENV2 NS2A, NS2B, NS4A, and NS4B are involved in suppressing RIG-I-directed antiviral response. NS2B binds to MAVS and IKKε and thus blocks RLR signaling (Fig. 5). These results further extend our knowledge of the innate immune evasion strategies utilized by DENV.
